# Association between Human Papillomavirus 16 Viral Load in Pregnancy and Preterm Birth

**DOI:** 10.3390/v16020298

**Published:** 2024-02-15

**Authors:** Pranamika Khayargoli, Marie-Hélène Mayrand, Joseph Niyibizi, François Audibert, Louise Laporte, Julie Lacaille, Ana Maria Carceller, Jacques Lacroix, Émilie Comète, François Coutlée, Helen Trottier

**Affiliations:** 1 Department of Social and Preventive Medicine, Université de Montréal, Montreal, QC H3N 1X9, Canada; pranamika.khayargoli@umontreal.ca (P.K.); marie-helene.mayrand@umontreal.ca (M.-H.M.); joseph.niyibizi@umontreal.ca (J.N.); 2 Centre de Recherche du Centre Hospitalier Universitaire Sainte-Justine, Université de Montréal, Montreal, QC H3T 1C5, Canada; 3 Centre de Recherche du Centre Hospitalier de l’Université de Montréal, Montreal, QC H2X 0A9, Canada; julie.lacaille.chum@ssss.gouv.qc.ca (J.L.); emilie.comete.chum@ssss.gouv.qc.ca (É.C.); 4 Department of Obstetrics and Gynecology, Université de Montréal, Montreal, QC H3C 3J7, Canada; 5 Department of Obstetrics and Gynecology, Centre Hospitalier Universitaire Sainte-Justine, Université de Montréal, Montreal, QC H3T 1C5, Canada; francois.audibert@umontreal.ca; 6 Department of Pediatrics, Centre Hospitalier Universitaire Sainte-Justine, Université de Montréal, Montreal, QC H3T 1C5, Canada; anacarceller@hotmail.com (A.M.C.); jlacroix052@gmail.com (J.L.); 7 Département de Microbiologie, Infectiologie et Immunologie, Université de Montréal, Montreal, QC H3T 1J4, Canada; francois.coutlee.med@ssss.gouv.qc.ca

**Keywords:** human papillomavirus (HPV), HPV16, viral load, pregnancy, preterm birth

## Abstract

Recent evidence shows increased preterm birth risk with human papillomavirus-16 (HPV16) infection during pregnancy. This study aimed to measure the association between HPV16 viral load during pregnancy and preterm birth. We used data from participants in the HERITAGE study. The Linear Array assay was used for HPV DNA testing on vaginal samples collected during the first and third trimesters of pregnancy. The HPV16 viral load was measured with a real-time polymerase chain reaction. We used logistic regression to measure the associations between HPV16 viral load during pregnancy and preterm birth (defined as birth before 37 weeks of gestation). The adjusted odd ratios (aORs) and the 95% confidence intervals [CIs] were estimated with inverse probability treatment weighting of the propensity score. This study included 48 participants who tested positive for HPV16 during the first trimester of pregnancy. The aOR for the association between first-trimester HPV16 viral load (higher viral load categorized with a cutoff of 0.5 copy/cell) was 13.04 [95% CI: 1.58–107.57]). Similar associations were found using different cutoffs for the categorization of viral load during the first and third trimesters. Our findings suggest a strong association between a high HPV16 viral load during pregnancy and preterm birth, demonstrating a biological gradient that reinforces the biological plausibility of a causal association.

## 1. Introduction

Preterm birth, usually defined as birth before 37 weeks of gestation, remains one of the leading causes of infant mortality and morbidity worldwide [1]. In 2020, 13.4 million infants were born prematurely, and complications from preterm birth led to almost 900,000 deaths in 2019 [1]. Although preterm birth survival proportions in high-income countries have risen in recent years [2], preterm births still account for nearly two-thirds of infant deaths in Canada [3]. Despite advances in obstetrics and neonatal research, the risk factors for a large proportion of preterm births remain unknown. A meta-analysis of 4.1 million births in five high-income countries reported that 65% of preterm births do not exhibit any identifiable risk factors [4]. Bacterial infections and inflammation are known risk factors for preterm delivery [5,6,7,8,9]. More recently, human papillomavirus (HPV) infection has been associated with preterm birth. Systematic reviews and meta-analyses have found an association between HPV and preterm birth [10,11,12]. We recently published results from a large cohort study, which included the detection of HPV genotypes individually at different timepoints during pregnancy. We reported a strong association between HPV16 persistence during pregnancy and preterm birth [13]. A decrease in the frequency of preterm births has also been identified in countries with successful HPV vaccination programs [14,15,16]. Although several studies suggest a potential role of HPV infection in preterm birth [10,11,13,17,18,19,20,21,22,23,24,25,26,27,28], and more specifically for HPV16 persistence [13], the biological mechanisms underlying this relationship remain unresolved. Showing a biological gradient between HPV16 infection and outcome would enhance biological plausibility and provide support for a causal relationship. Therefore, this study aimed to investigate the association between HPV16 viral load during pregnancy and preterm birth.

## 2. Material and Methods

### 2.1. Design and Participants

We used data from the HERITAGE (Human papillomavirus perinatal transmission and risk of HPV persistence among children) cohort study, whose design, methods, and results have previously been published [13,29,30,31]. The cohort included 1052 pregnant women, recruited between 2009 and 2016 from three academic hospitals in Montreal, Canada. Participants were eligible for the HERITAGE study if they were at least 18 years of age, pregnant at 14 weeks or earlier of gestation, able to provide written consent, and negative for HIV. The study was approved by the Human Research Ethics Committee of the Sainte-Justine University Health Center (protocol code: 2010265; date of approval: 12 March 2010). Written informed consent was obtained from all participants.

**Figure 1:**
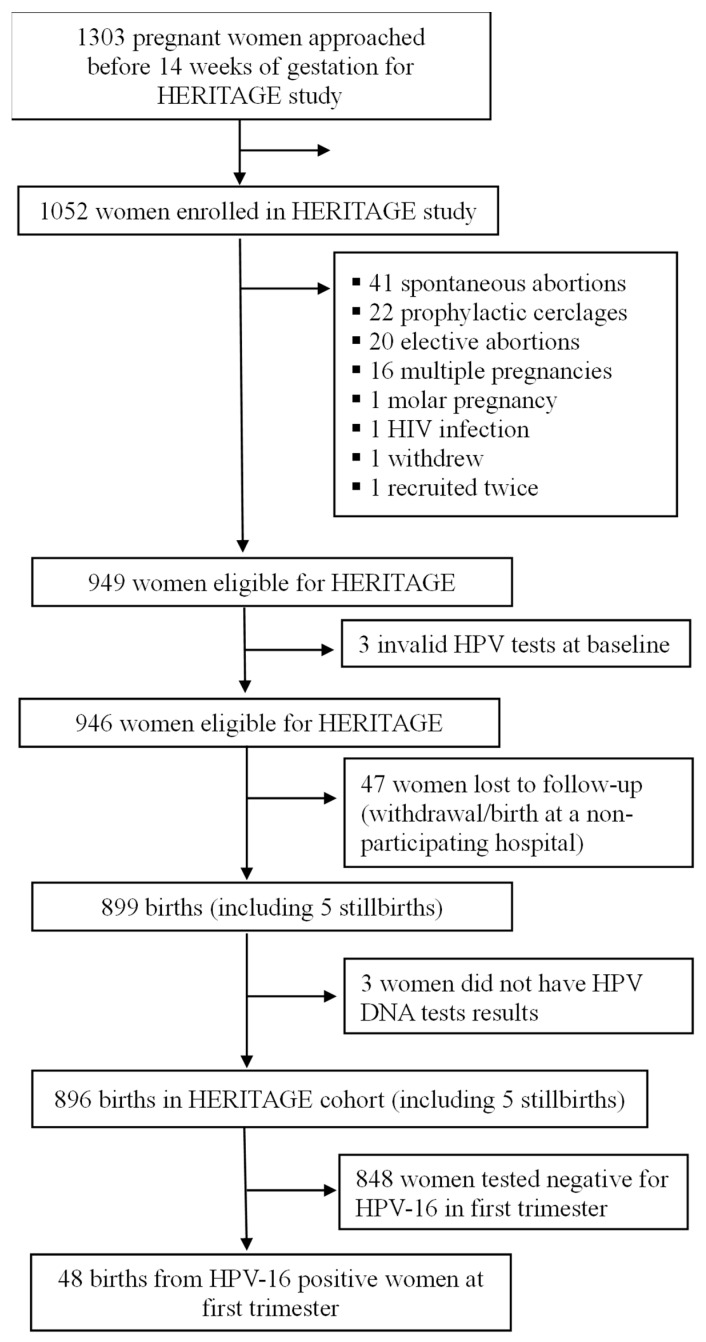
Study recruitment flowchart. HPV: human papillomavirus, HIV: human immunodeficiency virus. Figure was adapted from Niyibizi et al. [13].

In this analysis, we included participants if they had HPV16 detected at baseline (first-trimester visit). Participants were excluded if they had multiple pregnancies (twins or more), spontaneous or induced abortions or a history of cervico-isthmic insufficiency with prophylactic cerclage in the first trimester. A study flow diagram (Figure 1) presents the details of the 48 participants included in this analysis.

### 2.2. Sample Collection

The participants self-collected vaginal samples for genotype-specific HPV DNA testing at the recruitment visit (1st trimester of pregnancy) and at the third-trimester visit (32 to 35 weeks). Samples were processed as described previously [29].

### 2.3. HPV Testing

Extracted DNA from the vaginal samples was processed for HPV DNA detection and genotyping with the Linear Array HPV genotyping assay (Roche Molecular Systems, Branchburg, NJ, USA) [32].

The HPV16 viral loads were measured in HPV16-positive samples from the first- and third-trimester visits using real-time polymerase chain reaction (PCR) assays in a Light Cycler PCR and detection system (Roche Molecular Systems, Branchburg, NJ, USA) by measuring HPV16 and β-globin copy numbers in 2 µL of processed sample. The results were recorded as a crude number of copies as well as copy numbers per cell. Briefly, HPV16-positive samples were first screened for the presence of PCR inhibitors through the amplification of an internal control, as described previously [33]. All samples tested were shown to be free of inhibitor activity. HPV16 E6 DNA was quantified using a standard protocol [34]. The cycle thresholds obtained for each sample were compared to those of a titration curve obtained via serial 10-fold dilutions of the HPV16 DNA plasmid in 75 ng of human genomic DNA in 10 mM Tris-HCl (pH 8.2). The processed samples were then tested for the quantification of β-globin DNA to estimate the cell content of the samples [34]. The viral loads were calculated by dividing the number of HPV DNA copies by the total number of cells, which was estimated based on the number of β-globin copies.

### 2.4. Exposure, Outcome, and Covariates

The HPV16 viral load measured as copy numbers per cell was the exposure of interest. The outcome of interest was preterm birth, which was defined as a birth between 20 weeks and 0 days to 36 weeks and 6 days of gestation. The first-trimester ultrasound, which is part of routine prenatal care in the recruiting centers, was used to confirm gestational age based on menstrual period. One participant who underwent an emergency cerclage in the second trimester was considered to have experienced a spontaneous preterm birth although she ultimately delivered at 36 weeks of gestation.

Sociodemographic information, medical and sexual history, as well as alcohol and tobacco consumption were collected at recruitment, follow-up visits, and at birth using self-reported questionnaires. Information on pregnancy and delivery (labor onset, duration, date and time of membrane rupture and delivery, type of delivery) as well as medical history (history of preterm birth and cervical intraepithelial neoplasia treatment, gestational diabetes, hypertension, and urinary tract or genital infections) was extracted from the participants’ electronic medical records.

### 2.5. Statistical Analysis

The characteristics of the participants were described using means and standard deviations (SDs) or medians with quartiles (25th and 75th) for continuous variables and proportions (%) for categorical variables. The HPV16 viral loads measured in the 1st and 3rd trimesters were plotted in a line graph.

The association between HPV16 viral load and preterm birth was measured using logistic regression. Viral loads measured in the first and third trimesters were analyzed as a continuous variable and considered as binary variables using arbitrarily determined cutoffs of 0.5, 1, and 2 copies/cell (at above or below the cutoff), with the lowest viral load category being the referent in regression models. Crude odds ratios (ORs) and 95% confidence intervals (CIs) were computed. Adjusted ORs (aORs) (and 95% CIs) were estimated using propensity scores with inverse probability treatment weighting (IPTW). The propensity scores were estimated including potential confounders such as maternal age, ethnic origin (White or other), completed years of education, smoking at enrollment (yes or no), total days of use of alcohol since pregnancy (none, 1–4 days, or ≥5 days), history of preterm birth among parous women (yes or no), history of cervical intraepithelial neoplasia treatment (yes or no), and gestational diabetes (yes or no). Three variables had some missing values—smoking (1 out of 48 [2.1%]), history of cervical intraepithelial neoplasia treatment (6 out of 48 [12.5%]), gestational diabetes (2 out of 48 [4.2%])—that were imputed by the mode. The tests were two-sided, and the *p*-values were considered statistically significant at *p* < 0.05. Analyses were carried out using Stata/SE version 14.0.

## 3. Results

Table 1 summarizes the characteristics of the participants. Overall, the mean age (± SD) was 31.2 years (±4.7). Most of the participants were White (83.3%), had a university education (median of 17 completed years of education), did not smoke (89.6%), and did not have a history of preterm birth (94.7%) or of cervical intraepithelial neoplasia treatment (75%). At the first-trimester visit (*n* = 48), we found a mean HPV16 viral load of 1.63 copies/cell (SD = 5.64) (median = 8.0 × 10^−3^ copies/cell) with a maximum value of 31.46 copies/cell. Among these, 35 participants remained positive in the third trimester, with a mean HPV16 viral load (±SD) of 0.32 copies/cell (±0.97) (median = 5.29 × 10^−3^ copies/cell) and a maximum value of 5.07 copies/cell. Ten participants cleared their infection, and three had missing data on HPV status in the third trimester.

**Figure 2:**
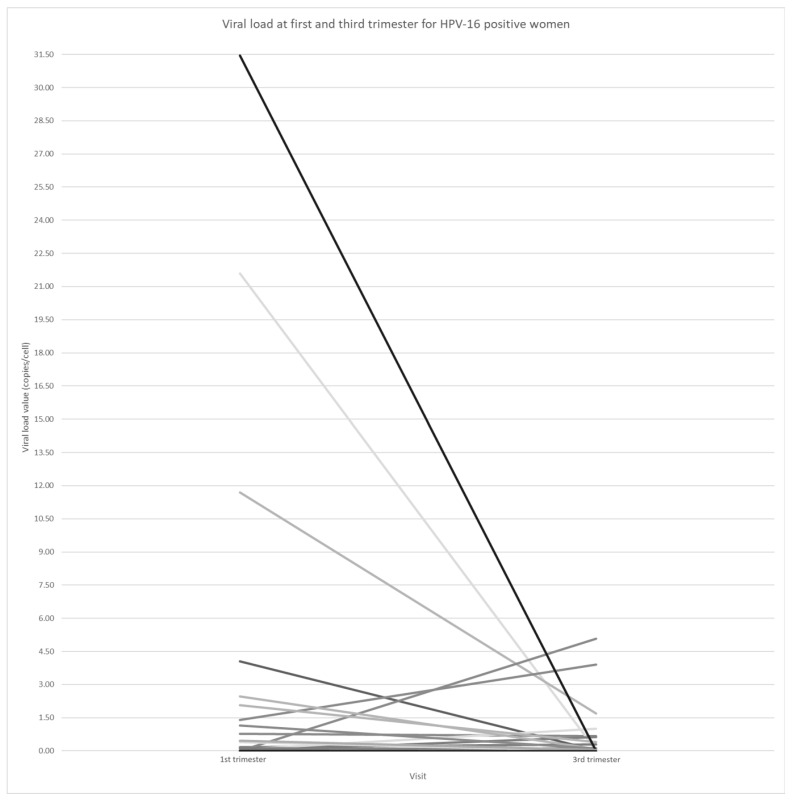
Viral load measured in the first and third trimester among HPV16-positive woman (*n* = 45; Among the 48 HPV16-positive pregnant women in the first trimester, 35 remained positive in the third trimester, 10 cleared their infection (viral load = 0), and 3 participants had missing HPV DNA testing results). HPV: human papillomavirus.

Figure 2 shows the HPV16 viral load values for each participant in the first and third trimester. Interestingly, we observed an overall decrease in the average viral load between the first and third trimester (paired *t*-test *p*-value = 0.0562). Five participants (10.4%) experienced preterm birth (four delivered at 36 weeks and one at 35 weeks), and among them, one was multiparous without a history of preterm birth. The five participants with preterm birth had a mean HPV16 viral load of 8.93 ± 13.51 copies/cell (median = 1.41 copies/cell and maximum value of 31.46 copies/cell) in the first trimester and remained positive for HPV16 in the third trimester, having 1.12 ± 1.72 copies/cell (median = 7.74 × 10^−3^ copies/cell and maximum value of 3.91 copies/cell). Only one participant with preterm birth (who gave birth at 36 weeks) had an increased viral load between the two trimesters while the others had decreased viral loads. Table 2 provides a description of the individual data on viral loads and other characteristics for each participant.

Table 3 shows the associations between HPV16 viral load and preterm birth. The viral load (as a continuous variable) in the first trimester was significantly associated with preterm birth; each unit increase in first-trimester viral load was associated with an increased risk of preterm birth by 13% (aOR [95% CI]: 1.13 [1.03–1.25]). When the viral load measures were dichotomized using a cutoff of 1 copy/cell, the highest viral load values measured in both the first and third trimester were associated with preterm birth with aORs of 15.03 [95% CI: 1.75–129.26] and 14.02 [95% CI: 1.28–153.48], respectively. Similar results were obtained for the other categorization, although they were not always statistically significant.

## 4. Discussion

We found significant associations between different measures of HPV16 viral load and preterm birth, albeit our small sample size (only 5 participants delivered prematurely among 48 participants positive for HPV16) warrants caution in drawing firm conclusions, despite strong ORs. When the viral load was analyzed as a continuous variable, we found that each unit increase in the HPV16 viral load in the first trimester increased preterm risk by 13% [95% CI: 3–25%]. When the viral load was analyzed dichotomously, participants with more than 1.0 copy/cell of HPV16 in the first trimester were 15.03 [95% CI: 1.75–129.26] more likely to experience preterm birth compared to participants who had less than 1.0 copy/cell. Similar results were found for viral loads measured in the third trimester.

Several studies have shown a positive association between HPV infection and preterm birth. A meta-analysis including 36 studies reported a pooled, age-adjusted OR of 1.50 [95% CI: 1.19–1.88] for the relationship between HPV and preterm birth [10]. The sensitivity analyses in this meta-analysis showed that this association was even stronger when restricting to studies of higher quality, such as those either using HPV testing (not cytology) or measuring HPV during pregnancy (not outside pregnancy). Other meta-analyses on the association between HPV and preterm birth reported pooled ORs of 2.84 [95% CI: 1.95–4.14] and of 2.12 [95% CI: 1.51–2.98] [11,12]. Most studies included in these meta-analyses had, however, not considered the potential role of individual HPV genotypes but either analyzed the role of any HPV genotypes (all genotypes combined together) or a cluster of genotypes. Recent studies have suggested an important role of HPV16 in this association but not for other HPV genotypes. A strong association between persistent HPV16 infection and preterm birth was recently found in our large cohort study [13]. This association was seen in our cohort for HPV16 only and not for any other individual genotype such as HPV31 or when HR-HPV were combined all together. A recent case-control study has also reported a significant association (*p* = 0.04) between HPV16 and preterm birth but not for other genotypes [28]. Population data from Australia, Finland, and Denmark also show a reduction in preterm births following the implementation of mass HPV vaccination programs, using vaccines targeting a limited number of genotypes, including HPV16 [14,15,16]. Although it remains very difficult to explain why the persistence (and viral load) of HPV16, and not of other genotypes, may be linked to preterm birth, our results suggest a biological gradient between HPV16 infection during pregnancy and risk of preterm birth, which enhances the biological plausibility of a causal relationship between HPV16 and prematurity.

To our knowledge, our study is the first to explore the impact of HPV16 viral load during pregnancy on preterm birth risk. We found a strong, albeit imprecise, association between HPV16 viral load and preterm birth. As the amount of extracellular virus can affect the inflammatory environment of the cervix, we also looked at the association between crude HPV16 copy number (per µL) and preterm birth, and the results were also similar although the ORs were somewhat attenuated. The current published literature on HPV viral load and persistence has mainly focused on clinical outcomes involving HPV-related precancerous lesions and cancers. A recent review of the literature [35] showed that a higher HPV16 viral load is associated with the severity of cervical preinvasive lesions while this is not usually found for other genotypes. It, therefore, appears plausible that the viral load of an HPV16 infection may play a role in other outcomes, such as preterm birth.

Our finding reinforces the plausibility of the link between HPV16 and preterm birth. However, the mechanism at the basis for the relationship is still unresolved. Specifically, two mechanisms for HPV infection’s role in preterm birth have been suggested [36]. Findings from in vitro studies suggest that HPV can alter trophoblast physiology and morphology with an increasing rate of apoptosis in the placenta, possibly causing abnormal placentation and compromised gestation [20,26,37,38]. HPV infection has also been suspected of disturbing the vaginal microbiome increasing its heterogeneity [39], which could in turn increase the production of pro-inflammatory cytokines and lead to early delivery. Nevertheless, it seems that the vaginal HPV viral load during pregnancy is an important parameter to consider. A higher viral load may cause greater cellular reactions in the cervix and disrupt the regular cellular pathways of parturition, which may either increase the risk of HPV transmission to the placenta or disrupt the vaginal microbiome during pregnancy and lead to preterm birth. On the other hand, a high viral load may be a marker of an immunologic dysfunction that may be linked to higher risk of preterm birth.

Our study has several strengths but also a few limitations. Given its prospective design, HPV DNA testing was conducted during pregnancy with repeated testing allowing the documentation of HPV persistence. A sensitive, type-specific HPV detection technique was used. The viral load was also measured with a specific and sensitive technique, considering the number of copies per cell, attenuating possible errors that may be caused by fluctuations in cell content. However, in the absence of data in the literature, we analyzed the impact of the viral load with different arbitrarily determined cutoffs, which do not necessarily have clinical justification. Additional studies are needed to confirm the role of HPV16 viral load in preterm birth and also to provide better rationale in categorizing viral load thresholds. The preterm birth estimates were also reliable given that the first-trimester ultrasound was routinely available. It is noteworthy that possible confounders were measured and adjusted for in our analysis using inverse probability treatment weighting (IPTW) with propensity scores, but we cannot exclude the possibility that there remains residual confounding because of unknown confounders or measurement errors.

## 5. Conclusions

This study is the first to date to explore the effect of HPV16 viral load during pregnancy and preterm birth and to suggest that high HPV16 viral loads during pregnancy are associated with preterm birth. This needs to be confirmed in other studies but may open new research avenues in the pathophysiology of idiopathic preterm birth. The presence of a biological gradient reinforces the biological plausibility of the link between HPV16 and preterm birth, although the exact mechanism behind this relationship remains to be demonstrated. Given that preterm birth remains a major health concern, it is important to better understand its etiology. If a causal relationship exists between HPV16 and preterm birth, mass HPV vaccination with the currently available vaccines will have a significant impact in reducing the number of preterm births globally.

## Figures and Tables

**Table 1:** Characteristics of HPV16-positive women in first trimester of pregnancy.

	Low HPV16 Viral Load (≤1 Copy/Cell) *n* = 40	High HPV16 Viral Load (>1 Copy/Cell) *n* = 8	Total Sample *n* = 48
Characteristics at baseline			
Mean age (SD); median [25–75%]	31.4 (4.6); 31 [28–34.5]	30.1 (5.5); 29 [26–33.5]	31.2 (4.7); 31 [28–34.5]
Completed years of education, median [25–75%]	17 [16–19]	17 [15.5–17]	17 [16–18.5]
Ethnicity, *n* (%)			
White	34 (85.0)	6 (75.0)	40 (83.3)
Arabic-West Asian	3 (7.5)	0	3 (6.3)
Native African	0	2 (25.0)	2 (4.2)
East Asian	1 (2.5)	0	1 (2.1)
Others ^a^	2 (5.0)	0	2 (4.2)
Smoker, *n* (%)			
Yes	3 (7.5)	1 (12.5)	4 (8.3)
No	36 (90.0)	7 (87.5)	43 (89.6)
Missing	1 (2.5)	0	1 (2.1)
Alcohol consumption (number of days since the beginning of pregnancy) ^b^, *n* (%)			
None	21 (52.5)	6 (75.0)	27 (56.3)
1–4	13 (32.5)	2 (25.0)	15 (31.3)
≥5	6 (15.0)	0	6 (12.5)
Nulliparous, *n* (%)			
Yes	25 (62.5)	4 (50)	29 (60.4)
No	15 (37.5)	4 (50)	19 (39.6)
History of preterm birth among parous women (*n* = 19), *n* (%)			
Yes	1 (6.7)	0	1 (5.3)
No	14 (93.3)	4 (100)	18 (94.7)
History of cervical intraepithelial neoplasia treatment, *n* (%)			
Yes	5 (12.5)	1 (12.5)	6 (12.5)
No	31 (77.5)	5 (62.5)	36 (75)
Missing	4 (10.0)	2 (25.0)	6 (12.5)
HPV16 viral load (copies/cell)			
Mean (SD)	5.9 × 10^−2^ (0.2)	9.5 (11.4)	1.6 (5.6)
Min–Max	4.0 × 10^−5^–0.77	1.15–31.46	4.0 × 10^−5^–31.46
Median [25–75%]	3.2 × 10^−3^ [6.9 × 10^−4^–3.8 × 10^−2^]	3.3 [1.7–16.6]	8.0 × 10^−3^ [1.2 × 10^−3^–0.1]
Characteristics during pregnancy			
Gestational diabetes, *n* (%)			
Yes	3 (7.5)	2 (25.0)	5 (10.4)
No	35 (87.5)	6 (75.0)	41 (85.4)
Missing	2 (5.0)	0	2 (4.2)
Pregnancy-induced hypertensive disorders, *n* (%)			
Yes	1 (2.5)	0	1 (2.1)
No	38 (95.0)	8 (100)	46 (95.8)
Missing	1 (2.5)	0	1 (2.1)
Urinary tract or genital infections ^c^, *n* (%)			
Yes	0	1 (12.5)	1 (2.1)
No	40 (100)	7 (87.5)	47 (97.9)
Pregnancy outcome, *n* (%)			
Preterm birth	2 (5.0)	3 (37.5)	5 (10.4)
Term birth	38 (95.0)	5 (62.5)	43 (89.6)

Note: Percentages may not sum to 100% due to rounding. HPV16 = human papillomavirus 16; SD = standard deviation. ^a^ Participants were categorized in the group “others” if they self-identified as being in two different ethnic groups. ^b^ Number of days on which there was consumption of at least one alcoholic drink since the start of pregnancy. ^c^ Urinary tract or genital infections include cystitis, bacterial vaginosis, active herpetic lesion, and non-specified urinary tract or genital infection.

**Table 2:** Viral load, preterm birth, and other characteristics of HPV16-positive women in the first trimester (*n* = 48).

Sequential Number	Age	1st-Trimester HPV16 Loads (Copies/Cell)	3rd-Trimester HPV16 Loads (Copies/Cell)	Difference in Viral Loads between 1st and 3rd Trimester	Preterm Birth	Gestational Age (Weeks)	History of Cervical Treatment *
1	28	11.69219	1.69494	−9.99725	Yes	36	No
2	40	1.40437	3.90897	2.5046	Yes	36	No
3	30	0.03511	0.00529	−0.02982	Yes	36	No
4	31	0.03989	0.00774	−0.03215	Yes	35	Missing
5	36	31.4574	0.00114	−31.45626	Yes	36	Yes
6	26	0.00284	0.00009	−0.00275	No	38	No
7	26	0.01402	0.00166	−0.01236	No	40	No
8	36	0.7735	0.65959	−0.11391	No	37	No
9	34	0.00049	0	−0.00049	No	39	No
10	22	0.05343	Missing	NA	No	38	No
11	26	21.59379	0.01448	−21.57932	No	39	No
12	31	0.09955	0.08503	−0.01451	No	38	No
13	36	0.00165	0.00589	0.00424	No	38	Yes
14	30	4.05012	0.02146	−4.02866	No	41	No
15	28	0.00579	Missing	NA	No	38	No
16	27	0.01189	0.00415	−0.00774	No	40	No
17	29	0.00051	Missing	NA	No	41	Missing
18	26	0.00279	0	−0.00279	No	38	No
19	26	0.00312	0	−0.00312	No	39	No
20	28	0.00025	0	−0.00025	No	39	Yes
21	33	0.00011	0.0011	−0.00099	No	40	Missing
22	30	0.16459	0.29646	0.13187	No	39	No
23	35	0.00036	0.00036	0	No	41	No
24	33	0.04063	0.00375	−0.03688	No	39	No
25	32	0.00134	5.06819	5.06686	No	39	No
26	28	0.00004	0.0004	0.00036	No	39	No
27	33	0.05176	1.00443	0.95267	No	40	No
28	38	0.00015	0	−0.00015	No	38	Missing
29	33	0.00486	0.62714	0.62228	No	40	No
30	26	0.02287	0.01027	−0.0126	No	40	No
31	26	2.46677	0.08264	−2.38413	No	39	Missing
32	24	1.14868	0.10666	−1.04202	No	40	No
33	47	0.00065	0	−0.00065	No	39	No
34	31	0.00327	0.00133	−0.00194	No	40	No
35	31	0.00108	0.01091	0.00983	No	41	Yes
36	32	0.12291	0.04096	−0.08195	No	39	No
37	30	0.00473	0.01793	0.0132	No	37	No
38	31	2.07335	0.38704	−1.6863	No	40	Missing
39	33	0.00063	0	−0.00063	No	40	No
40	29	0.01605	0.12466	0.10861	No	40	No
41	29	0.00227	0	−0.00227	No	39	No
42	32	0.39554	0.00759	−0.38795	No	39	No
43	38	0.45647	0.03041	−0.42606	No	41	Yes
44	35	0.00308	0.00004	−0.00304	No	39	No
45	36	0.0001	0	−0.0001	No	40	No
46	36	0.01013	0	−0.01013	No	41	No
47	35	0.00072	0.00524	0.00451	No	41	Yes
48	26	0.00307	0.00062	−0.00244	No	39	No

* Most cervical treatments in Canada are LEEP. HPV16 = human papillomavirus 16; NA = not available.

**Table 3:** Odds ratio for associations between HPV16 viral load and preterm birth.

HPV16 Viral Load (Number of Copies/Cell)		Odds Ratio (95% CI)	
	Number of Preterm Births/Total Women	Crude	Adjusted ^c^
Viral load in the first trimester (continuous)	5/48 ^a^	**1.15 (1.01–1.31)**	**1.13 (1.03–1.25)**
Viral load in the third trimester (continuous)	5/45 ^b^	1.75 (0.90–3.41)	1.84 (0.80–4.23)
First trimester			
Low viral load (≤0.5 copies/cell)	2/39	Referent	Referent
High viral load (>0.5 copies/cell)	3/9	**9.25 (1.27–67.42)**	**13.04 (1.58–107.57)**
Third trimester			
Low viral load (≤0.5 copies/cell)	3/39	Referent	Referent
High viral load (>0.5 copies/cell)	2/6	6.00 (0.76–47.36)	6.75 (0.76–59.67)
First trimester			
Low viral load (≤1 copy/cell)	2/40	Referent	Referent
High viral load (>1 copy/cell)	3/8	**11.40 (1.52–85.73)**	**15.03 (1.75–129.26)**
Third trimester			
Low viral load (≤1 copy/cell)	3/41	Referent	Referent
High viral load (>1 copy/cell)	2/4	**12.67 (1.29–124.51)**	**14.02 (1.28–153.48)**
First trimester			
Low viral load (≤2 copies/cell)	3/42	Referent	Referent
High viral load (>2 copies/cell)	2/6	6.50 (0.83–51.20)	6.24 (0.66–59.06)
Third trimester			
Low viral load (≤2 copies/cell)	4/43	Referent	Referent
High viral load (>2 copies/cell)	1/2	9.75 (0.51–187.53)	14.67 (0.72–300.70)

Bold values represent statistically significant results; HPV16 = human papillomavirus 16. ^a^ Total number of women with HPV16 DNA infection in the first trimester of pregnancy. ^b^ Total number of HPV16 DNA infections in the third trimester of pregnancy (*n* = 45, excluding 3 participants with HPV16 infection in the first trimester of pregnancy who had missing HPV DNA testing results in the third trimester). ^c^ Adjusted estimates obtained using propensity-score-based inverse probability treatment weights including the following variables: maternal age (years; continuous); ethnic origin (White or other); completed education (years; continuous); smoking at enrollment (yes or no); total days with use of alcohol since pregnancy (none, 1–4 days, or ≥5 days); history of preterm birth among parous women (yes or no); history of cervical intraepithelial neoplasia treatment (yes or no); which consisted of 1 ablative treatment, 6 excisional treatments and 1 unknown; and gestational diabetes (yes or no).

## Data Availability

The raw data supporting the conclusions of this article will be made available by the authors on request.
